# The Antimalarial Chloroquine Suppresses LPS-Induced NLRP3 Inflammasome Activation and Confers Protection against Murine Endotoxic Shock

**DOI:** 10.1155/2017/6543237

**Published:** 2017-02-22

**Authors:** Xiaoli Chen, Ning Wang, Yuanfeng Zhu, Yongling Lu, Xin Liu, Jiang Zheng

**Affiliations:** Medical Research Center, Southwest Hospital, Third Military Medical University, Chongqing 400038, China

## Abstract

Activation of the NLRP3 inflammasome, which catalyzes maturation of proinflammatory cytokines like IL-1*β* and IL-18, is implicated and essentially involved in many kinds of inflammatory disorders. Chloroquine (CQ) is a traditional antimalarial drug and also possesses an anti-inflammatory property. In this study, we investigated whether CQ suppresses NLRP3 inflammasome activation and thereby confers protection against murine endotoxic shock. CQ attenuated NF-*κ*B and MAPK activation and prohibited expression of IL-1*β*, IL-18, and* Nlrp3* in LPS treated murine bone marrow-derived macrophages (BMDMs), demonstrating its inhibitory effect on the priming signal of NLRP3 activation. Then, CQ was shown to inhibit caspase-1 activation and ASC specks formation in BMDMs, which indicates that CQ also suppresses inflammasome assembly, the second signal for NLRP3 inflammasome activation. In a murine endotoxic shock model, CQ effectively improved survival and markedly reduced IL-1*β* and IL-18 production in serum, peritoneal fluid, and lung tissues. Moreover, CQ reduced protein levels of NLRP3 and caspases-1 p10 in lung homogenates of mice with endotoxic shock, which may possibly explain its anti-inflammatory activity and life protection efficacy in vivo. Overall, our results demonstrate a new role of CQ that facilitates negative regulation on NLRP3 inflammasome, which thereby confers protection against lethal endotoxic shock.

## 1. Introduction

Inflammation is a fundamental host protective response towards external or internal stimuli. However, uncontrolled inflammation, as characterized by excessive cytokine release, also contributes to a plenty of acute and chronic clinical disorders, including obesity, cardiovascular diseases, tumor, and sepsis [1–3]. IL-1*β* and IL-18 are two proinflammatory members of the interleukin-1 (IL-1) superfamily which function as “master” cytokines in the complex network of inflammatory responses [4–6]. In different septic murine models, IL-1*β* and IL-18 inhibition is consistently effective for protection against inflammation and septic shock [7]. Additionally, IL-1*β* and IL-18 are unique as they are controlled at both transcriptional and posttranscriptional levels. Transcription of the precursor of IL-1*β* and IL-18 is induced by stimulation from extracellular signals and activation of downstream signal pathway (other proinflammatory mediators). Moreover, secretion of bioactive IL-1*β* and IL-18 requires activation of inflammasome, a multiprotein oligomer assembled to catalyze the splicing of the IL-1*β* and IL-18 into mature forms.

The NACHT, LRR, and PYD domains-containing protein 3 (NLRP3) inflammasome is one of the most crucial inflammasomes that controls the maturation of IL-1*β* and IL-18 [8]. In response to pathogen associated molecular patterns (PAMPs) and damage associated molecular patterns (DAMPs), activation of NLRP3 leads to the formation of a cytosolic multimeric protein complex composed of apoptosis-associated speck-like protein containing CARD (ASC) and cleaved caspase-1. Activated caspase-1 subsequently functions to cleave the proinflammatory IL-1 family of cytokines into their mature forms, IL-1*β* and IL-18, and cause cell pyroptosis [9, 10]. Two signals are needed for NLRP3 inflammasome activation. The first signal is the priming signal involving mainly the TLR/NF-*κ*B pathway which upregulates the expression of NLRP3 and pro-IL-1 [11]. Bacterial lipopolysaccharide (LPS) is the most commonly used inducer for this signal. The second signal is the activation signal, which regulates potassium efflux and ASC speck formation, initiates assembly of protein complex containing NLRP3, ASC, and procaspase-1, and triggers splicing of caspase-1 into its active form. ATP and nigericin are common stimulators for this signal. In addition to triggering the production of IL-1*β* and IL-18, excessive inflammasome activation also mediates cellular pyroptosis, which ultimately destroys the innate and also impairs adaptive immunity [12, 13]. Therefore, it represents an important therapeutic strategy for inflammation control by interfering with the activation of NLRP3 inflammasome and suppressing the production of IL-1*β* and IL-18 [7, 14, 15].

CQ is a lysosomotropic weak base compound and is traditionally used as an antimalarial agent [16]. Several previous studies, including one of our former works, have shown that CQ possesses anti-inflammatory activity by effectively inhibiting production of proinflammatory cytokines in monocytes/macrophages in response to LPS stimulation [17, 18]. However, most of these studies investigated the anti-inflammatory effects of CQ by analyzing its modulation of TLR4 dependent signaling pathways. It remains unclear whether CQ could affect the NLRP3 inflammasome activation and thereby interferes with IL-1*β* and IL-18 production and maturation. Herein, we examined the inhibitory effect of CQ on IL-1*β* and IL-18 production in murine bone marrow-derived macrophages (BMDMs). We also investigated the underlying mechanism by detecting whether CQ suppressed NLRP3 inflammasome activation and thereby inhibited IL-1*β* and IL-18 secretion. We further explored the inhibitory activities of CQ against NLRP3 inflammasome in a murine endotoxic shock model which may possibly explain its anti-inflammatory and life-protective effects.

## 2. Materials and Methods

### 2.1. Animals

BALB/c mice were obtained from HFK Bioscience (Beijing, China). Mice were housed under a 12 h day/night cycle under specific pathogen-free conditions with temperatures ranging between 22 and 26°C and relative humidity of 40–70%. All animal experiments were approved by the ethical committee of the Third Military Medical University for animal care and use.

### 2.2. Cells Preparation

BMDMs were derived from bone marrow cells of BALB/c murine mice (6–8 weeks) as previously reported. Three murine mice were sacrificed by cervical dislocation. Then, legs and abdomens were sterilized with iodophor and muscles were removed to expose the bones, and then bones were cut at both ends and rinsed with saline. Cells in the bone marrow were collected and cultured in endotoxin-free Dulbecco's modified Eagle's medium (DMEM) with 10% fetal bovine serum (SERA, Germany) and 20 ng/ml M-CSF (Sigma, USA), in 5% CO_2_ at 37°C. Nonadherent cells were discarded. BMDMs were cultured for at least 6 days to ensure full differentiation.

### 2.3. Cell Treatment and LDH Release Detection

Cells (1 × 10^6^ cells/ml) were seeded on culture plates in DMEM with 10% fetal bovine serum and allowed to complete adherence. Then, cells were pretreated with CQ (Sigma, USA) for 2 h at the indicated concentrations and then primed with* Escherichia coli* 055:B5 LPS (Sigma, USA), Pam3CSK4 (Invivogen, USA), or R848 (Invivogen, USA) for the indicated time, followed by treatment with ATP or nigericin (Sigma, USA) for 1 h to 4 h. Supernatant and cell lysates were collected for further assays. Cytotoxicity was measured by detection of lactate dehydrogenase (LDH) release in the supernatant using the CytoTox96 nonradioactive cytotoxicity assay (Promega, USA) according to the manufacturer's instructions.

### 2.4. Enzyme-Linked Immunosorbent Assay (ELISA) for Cytokines

IL-1*β* and IL-18 in cell-culture supernatants, cell lysates, serum, or lung homogenates were detected with ELISA kits (eBioscience, USA) according to the manufacturer's instructions.

### 2.5. Western Blot Analysis

Cells (2 × 10^6^ cells/dish) were plated in dishes with DMEM. Cells were then lysed with M-PER Mammalian Protein Extraction Reagent (Thermo Fisher, Rockford, USA) supplemented with complete protease inhibitors and PhosSTOP phosphatase inhibitor (Roche, Germany). Total protein concentration in the extracts was measured by BCA protein assay kit (Thermo Fisher, USA). Equal amounts of protein per sample were separated by SDS-PAGE and then transferred onto PVDF membranes. The membrane was blocked with 5% BSA in PBST and incubated with corresponding primary antibodies overnight at 4°C, followed by incubation with corresponding HRP-conjugated secondary antibodies. Primary antibodies include NF-*κ*B p65 and phospho-NF-*κ*B p65 (Ser536), MAPK Family Antibody Sampler Kit and Phospho-MAPK Family Antibody Sampler Kit (Cell Signaling Technology, USA), caspase-1, I*κ*B-alpha, and phospho-I*κ*B-alpha (S36) (Abcam, UK), NLRP3/NALP3 (R&D Systems, USA), ASC (Novus, USA), and caspase-1 p10, GAPDH, and tubulin (Santa Cruz Biotechnology, USA). The bands were detected using SuperSignal West Femto Chemiluminescent Substrate (Thermo Fisher, Rockford, USA) according to the manufacturer's instruction. The target protein expression levels were quantitated by measuring band intensities using “ImageJ” software. The results were normalized to tubulin or GAPDH.

### 2.6. qRT-PCR

Total RNA was extracted with TRIzol reagent (Tiangen, China) according to the manufacturer's protocol. The mRNA was then transcribed into cDNA using ReverTra Ace® qRT-PCR Kit (Toyobo, Japan). The cDNA abundance was measured by quantitative real-time PCR (qRT-PCR) using SYBR® Green Real-Time PCR Master Mix on CFX96 Touch™ Real-Time PCR Detection System (Bio-Rad, USA). Then, qRT-PCR was performed to quantitate the mRNA of* Nlrp3*, IL-1*β*, IL-18, and actin. The sequences of primers used for PCR were as follows: 5′-ATCAACAGGCGAGACCTCTG-3′ and 5′-GTCCTCCTGGCATACCATAGA-3′ for* Nlrp3*; 5′-GAAATGCCACCTTTTGACAGTG-3′ and 5′-TGGATGCTCTCATCAGGACAG-3′ for IL-1*β*; 5′-GACTCTTGCGTCAACTTCAAGG-3′ and 5′-CAGGCTGTCTTTTGTCAACGA-3′ for IL-18; 5′-ACAAGGCACGGGACCTATG-3′ and 5′-TCCCAGTCAGTCCTGGAAATG-3′ for caspase-1; 5′-GGGAAATCGTGCGTGACATCAAAG-3′ and 5′-CATACCCAAGAAGGAAGGCTGGAA-3′ for *β*-actin. Primer specificity was determined by melt curve analysis. Actin was used as the endogenous control to estimate other genes. The results are presented as fold induction over levels in untreated control cells. Relative gene levels were calculated according to the comparative Ct method formula 2^−ΔΔCt^.

### 2.7. Immunofluorescence

Immunofluorescence assay was carried out as described previously. Briefly, cells (1 × 10^5^/ml) were plated on glass-bottom cell-culture dishes (NEST, China) overnight for adherence, fixed in 4% paraformaldehyde solution, permeabilized in 0.2% Triton X-100, and blocked in 3% BSA at RT for 1 h. Cells were incubated in diluted antibody for NF-*κ*B p65 (1 : 200) and ASC (1 : 100) overnight at 4°C and then incubated in cy3-labeled diluted secondary antibody (1 : 400) and the nucleus was stained by DAPI. Images were obtained using Zeiss LSM780 fluorescent confocal microscopy (Carl Zeiss, Germany). At least five distinct fields were analyzed from each treatment for calculating the percentage of cells containing ASC speck.

### 2.8. Animal Model of Endotoxic Shock

Endotoxic shock was induced in BALB/c murine models (males, 7–9 weeks, 20–25 g) by intravenous injection of LPS (15 mg/kg), as previously described. CQ was administered before LPS injection at the indicated doses. The number of deaths was recorded at the corresponding time for 7 days. Blood was collected at 6 h after LPS injection and then centrifuged for 10 min at 2500*g* to isolate serum. Lung tissue was weighed and homogenized in Tissue Protein Extraction solution and centrifuged for 10 min at 12,000*g*. The tissue homogenate and serum were stored at −80°C before analysis.

### 2.9. Statistical Analysis

The results were presented as means ± SEM of at least three repeating times. Statistical analysis was performed using GraphPad Prism 6.0 (GraphPad Software Inc., San Diego, CA). Analysis was performed using Student's *t*-test or one-way ANOVA. Values of *P* < 0.05 were considered to be statistically significant.

## 3. Results

### 3.1. CQ Suppresses IL-1*β* and IL-18 Secretion in LPS Stimulated BMDMs

**Figure 1:**
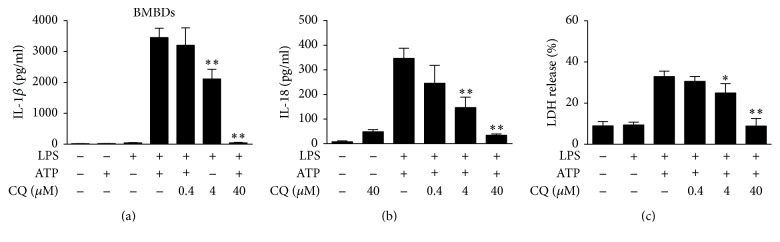
CQ suppress IL-1*β* and IL-18 secretion in LPS- and ATP-primed BMDMs. BMDMs were pretreated with or without 0.4, 4, and 40 *μ*M CQ for 1 h and then stimulated with LPS (100 ng/ml) for 4 h and ATP (2 mM) for an additional 1 h. The IL-1*β* and IL-18 levels in culture supernatants were determined by ELISA (*n* = 3). ^*∗∗*^*P* < 0.01 versus LPS plus ATP group (a-b). LDH release from BMDMs was measured by LDH assay (c). ^*∗*^*P* < 0.05, ^*∗∗*^*P* < 0.01 versus LPS plus ATP group. All data were expressed as mean ± SEM in triplicate and are representative of three independent experiments.

To test whether CQ affected the production of inflammasome controlled cytokines, we detected IL-1*β* and IL-18 levels in the supernatants of murine BMDMs. Cells stimulated by LPS or ATP alone did not produce IL-1*β* or IL-18. Cotreatment with ATP significantly increased supernatant IL-1*β* and IL-18 in LPS-primed BMDMs. Pretreatment with CQ dose-dependently inhibited the release of IL-1*β* (Figure 1(a)) and IL-18 (Figure 1(b)) in response to LPS and ATP. In the cell viability assay, CQ treatment did not increase LDH release in BMDMs 1 h after ATP treatment (Figure S2 in the Supplementary Material available online at https://doi.org/10.1155/2017/6543237). However, marked elevation of LDH release was detected 2 h after ATP treatment, indicating pyroptosis formation. CQ significantly reduced LDH release and thereby inhibited pyroptosis in BMDMs (Figure 1(c)). Supernatant IL-1*β* was similarly inhibited by CQ in murine peritoneal macrophages stimulated by LPS and ATP (Figure S1). In addition, CQ also decreased IL-1*β* release in Pam3CSK4 or R848 stimulated BMDMs, indicating a general inhibitory effect on different TLRs activation (Figure S3).

### 3.2. CQ Inhibits Transcription of IL-1*β* and IL-18 in LPS Stimulated BMDMs

**Figure 2:**
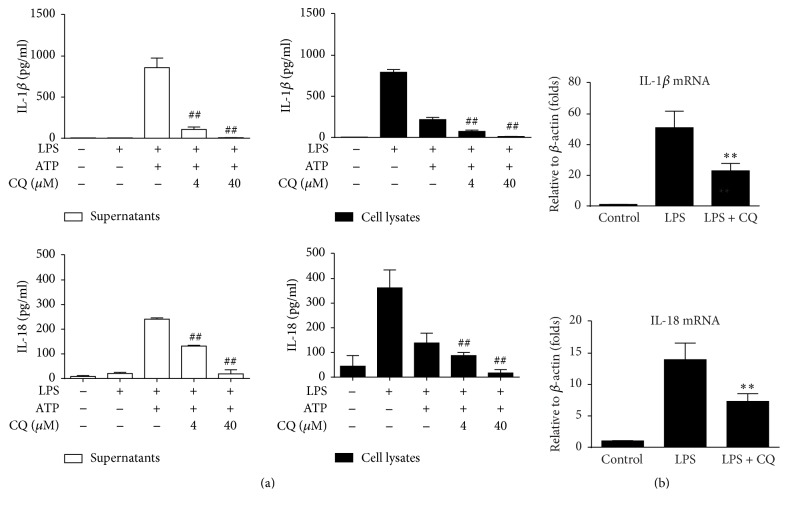
CQ inhibits transcription and maturation of IL-1*β* and IL-18. (a) BMDMs were pretreated with CQ (4, 40 *μ*M) for 1 h, primed with LPS (100 ng/ml) for 4 h, and stimulated with ATP (2 mM) for an additional 1 h. Cell lysates and supernatants were prepared and IL-1*β* level was detected by ELISA. ^*∗∗*^*P* < 0.01 versus LPS plus ATP group. (b) IL-1*β* and IL-18 mRNA expressions were measured by qRT-PCR. BMDMs were pretreated with CQ (40 *μ*M) for 1 h and then primed with LPS (100 ng/ml) for 2 h. Data are shown as the means ± SEM (*n* = 3). ^*∗∗*^*P* < 0.01 versus LPS group; ^##^*P* < 0.01 versus LPS plus ATP group.

To further investigate how CQ affected the production of IL-1*β* and IL-18, we detected levels of pro-IL-1*β* (cell lysates) and mature IL-1*β* (culture supernatants). As shown in Figure 2(a), LPS treatment alone increased intracellular IL-1*β* production but did not enhance the release of IL-1*β*. ATP cotreatment induced IL-1*β* maturation and thereby caused the decrease of cellular IL-1*β* and increased IL-1*β* release in the supernatants. CQ inhibited both pro-IL-1*β* production and mature IL-1*β* secretion in LPS- and ATP-primed BMDMs. Consistent effects were also observed in IL-18 detection (Figure 2(a)). To investigate whether CQ affected transcription of cytokines upon LPS stimulation, the mRNA expression of pro-IL-1*β* and pro-IL-18 was measured by qRT-PCR. Pretreatment of CQ significantly decreased pro-IL-1*β* and IL-18 mRNA expression (Figure 2(b)). These results suggested that CQ might suppress the production of IL-1*β* and IL-18 at both transcriptional and posttranscriptional levels. We also performed additional study by selectively adding CQ at the stage of either LPS priming or ATP treatment. Although CQ attenuated IL-1*β* release when added at both stages, it demonstrated stronger activity when cotreated with LPS (Figure S4).

### 3.3. CQ Inhibits the Activation of NF-*κ*B and MAPK Signaling Pathways

**Figure 3:**
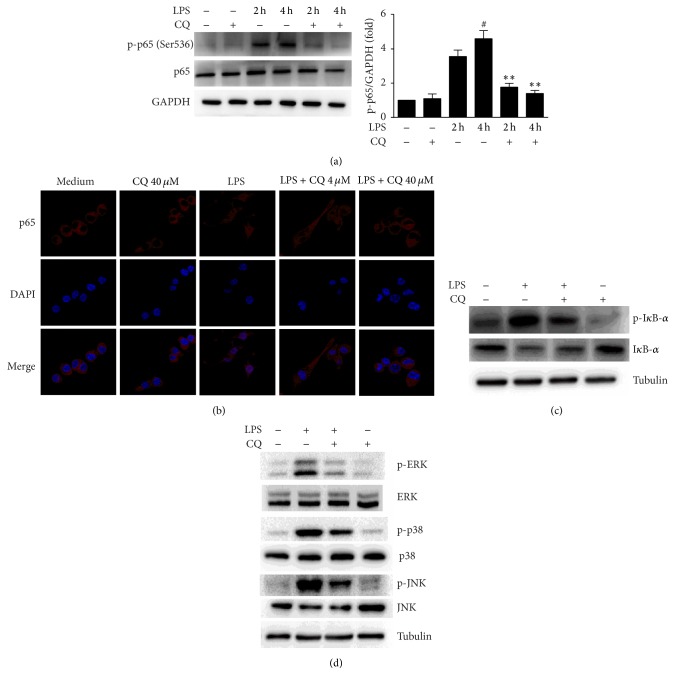
CQ restrains NF-*κ*B p65 phosphorylation and nuclear localization. (a) Western blot of phospho-NF-*κ*B p65 (Ser536). Cells were pretreated with 40 *μ*M CQ for 1 h and then 100 ng/ml LPS treated for 2 h and 4 h, respectively. Total cell protein was collected for western blot of NF-*κ*B p65 phosphorylation and GAPDH. ^#^*P* < 0.05 versus LPS group (2 h). ^*∗∗*^*P* < 0.01 versus LPS group (4 h). (b) Cells were pretreated with 40 *μ*M CQ for 1 h and then stimulated by LPS for 30 min. I*κ*B-*α* and phospho-I*κ*B-*α* levels were detected by western blot. (c) Confocal images of nuclear translocation of NF-*κ*B p65. Cells were pretreated with different concentrations of CQ for 1 h, followed by 100 ng/ml LPS treatment for 2 h. Cells were stained by anti-NF-*κ*B p65 (red) antibodies and DAPI (blue) for nuclear staining. (d) Cells were treated as in (b). ERK, p38, and JNK and their phosphorylated forms (p-I*κ*B-*α*, p-ERK, p-p38, and p-JNK) in cell lysates were detected by western blot.

Recognition of LPS by TLR4 initiates signaling pathways involving activation of NF-*κ*B and MAPK, which also play critical roles in the transcriptional upregulation of IL-1*β* and IL-18 [19, 20]. In our study, we detected enhanced expression levels of p-NF-*κ*B p65 (Ser536) after LPS treatment, which was significantly inhibited by CQ (Figure 3(a)). We further detected I*κ*B-*α* expression by LPS stimulation in BMDMs and observed that CQ consistently inhibited the phosphorylation and degradation of I*κ*B-*α* (Figure 3(b)). Consistent with the p-NF-*κ*B p65 detection, a dramatic increase of p65 nuclear translocation was found in BMDMs treated by LPS for 4 h, which was also suppressed by CQ pretreatment dose-dependently (Figure 3(c)). Moreover, CQ also inhibited the activation of ERK, p38, and JNK in LPS stimulated BMDMs (Figure 3(d)). These results indicate that the inhibitory effect of CQ on the transcription of IL-1*β* and IL-18 may involve the downregulation of NF-*κ*B and MAPK signaling.

### 3.4. CQ Inhibits Transcriptional Expression of NLRP3 and Suppresses the Activity of NLRP3 Inflammasome

**Figure 4:**
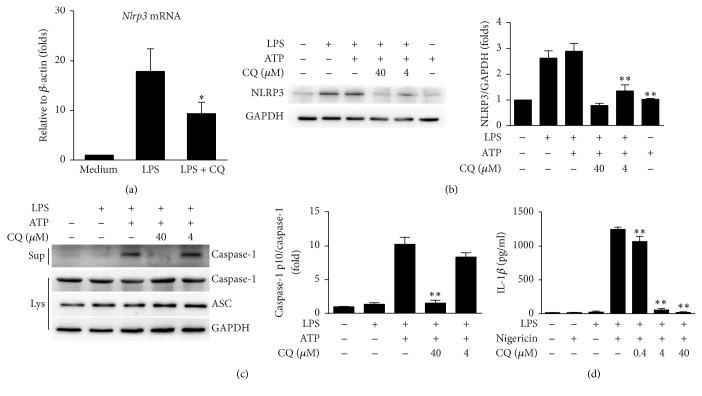
CQ dampens NLRP3 inflammasome activation. BMDMs were pretreated with CQ (4, 40 *μ*M) for 1 h and then stimulated with LPS (100 ng/ml) for 4 h and ATP (2 mM) for an additional 1 h. (a)* Nlrp3* mRNA expression was measured by qRT-PCR. (b) NLRP3 and GAPDH were measured in cell lysates by western blot. (c) Western blot analysis of cleaved caspase-1 (p10) in culture supernatants (Sup) or of procaspase-1, ASC, and tubulin in cell lysates (Lys). (d) BMDMs were pretreated with CQ at the indicated concentration for 1 h and then stimulated with LPS (100 ng/ml) for 4 h and nigericin (10 *μ*M) for 30 min. Production of mature IL-1*β* in supernatants was measured by ELISA. Data are shown as means ± SEM; ^*∗*^*P* < 0.05, ^*∗∗*^*P* < 0.01 versus LPS plus ATP group.

A precondition of NLRP3 inflammasome activation is the transcriptional upregulation of* Nlrp3*, which requires the transduction of NF-*κ*B signaling [21, 22]. Therefore, we speculated that CQ might thus interfere with the expression of nlrp3. Our results showed that CQ decreased* Nlrp3* mRNA significantly in LPS stimulated BMDMs (Figure 4(a)). Moreover, the protein levels of NLRP3 were also upregulated by LPS or cotreatment of LPS and ATP, while CQ consistently inhibited the elevated NLRP3 expression (Figure 4(b)). In addition to NLRP3 upregulation, the activation of the NLRP3 inflammasomes is also characterized by cleavage of caspases-1. As shown by immunoblotting, costimulation with LPS and ATP resulted in an elevated level of caspases-1 p10, the active form of caspases-1. CQ pretreatment markedly downregulated caspases-1 p10 levels without altering the expression of procaspase-1 and ASC (Figure 4(c)). To fully determine that CQ suppressed NLRP3 inflammasome activation, we used another NLRP3 agonist nigericin to replace ATP and observed similar inhibitory effects of CQ on IL-1*β* release in BMDMs (Figure 4(d)). These data indicated that CQ could also interfere with the maturation of IL-1*β* and IL-18 by inhibiting NLRP3 inflammasome activation.

### 3.5. CQ Restrains ASC Speck Formation and Potassium Efflux

**Figure 5:**
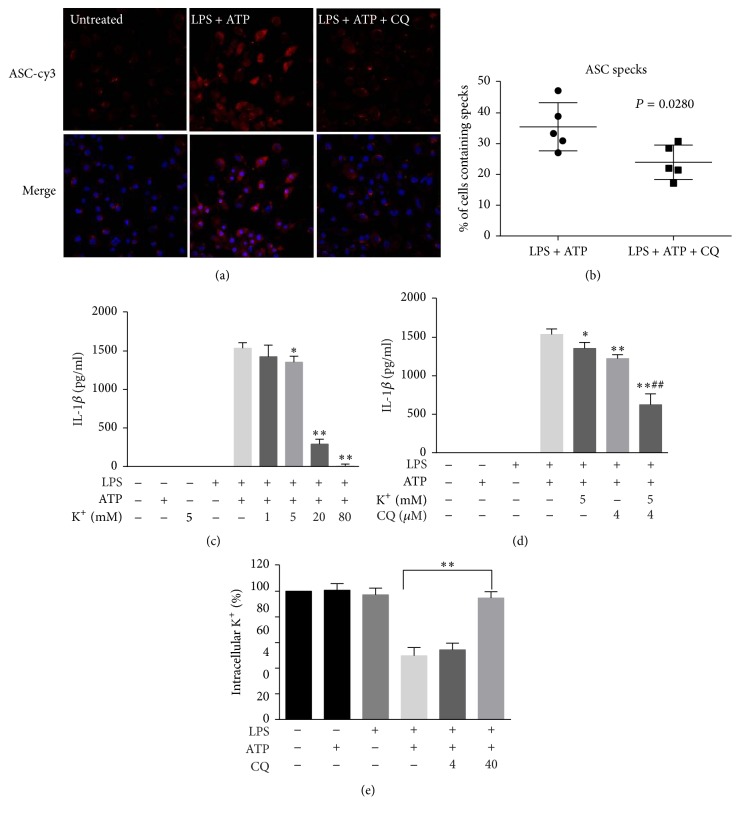
CQ restrains ASC speck formation and potassium efflux. (a) Representative immunofluorescence images of ASC speck formation in LPS-primed BMDMs stimulated with ATP in the presence or absence of CQ (20 *μ*M) were acquired by confocal microscopy. (b) A percentage of cells containing specks were quantified in at least five distinct fields. (c, d) LPS-primed BMDMs were treated for 1 h with 2 mM ATP in a medium containing the specified [K^+^], and the secreted IL-1*β* was measured. (e) CQ pretreatment BMDMs were treated with LPS plus ATP, and the intracellular content of K^+^ was detected. Data are shown as means ± SEM; ^##^*P* < 0.01 versus CQ pretreatment (4 *μ*M) group; ^*∗*^*P* < 0.05, ^*∗∗*^*P* < 0.01 versus LPS plus ATP group.

Large intracellular ASC aggregates are called ASC specks in the cytosol and must be formed when NLRP3 inflammasome is assembled and activated. As demonstrated by immunofluorescence, the ASC specks were condensed in BMDMs upon LPS and ATP stimulation. Pretreatment with CQ markedly attenuated the ASC specks formation induced by ATP, suggesting that CQ could also restrain the formation of NLRP3 complex (Figures 5(a) and 5(b)). Potassium efflux is a common step that is essential for NLRP3 inflammasome activation [23]. In our study, treatment with extracellular [K^+^] in cultured BMDMs caused decreased release of IL-1*β* upon LPS and ATP treatment (Figure 5(c)). Moreover, combined use of 4 *μ*M CQ and 5 mM extracellular K^+^ further decreased IL-1*β* release as compared with treatment with extracellular K^+^ or CQ alone (Figure 5(d)). We next directly detected intracellular [K^+^]. Results showed that CQ reversed the decline of intracellular [K^+^] elicited by LPS and ATP treatment (Figure 5(e)). Our data suggest that CQ blocks NLRP3 inflammasome activation both by reducing K^+^ efflux from BMDMs and by inhibiting ASC specks formation to the assembly of the NLRP3 inflammasome.

### 3.6. CQ Injection Protects against Murine Endotoxic Shock by Suppressing Activation of the NLRP3 Inflammasome

**Figure 6:**
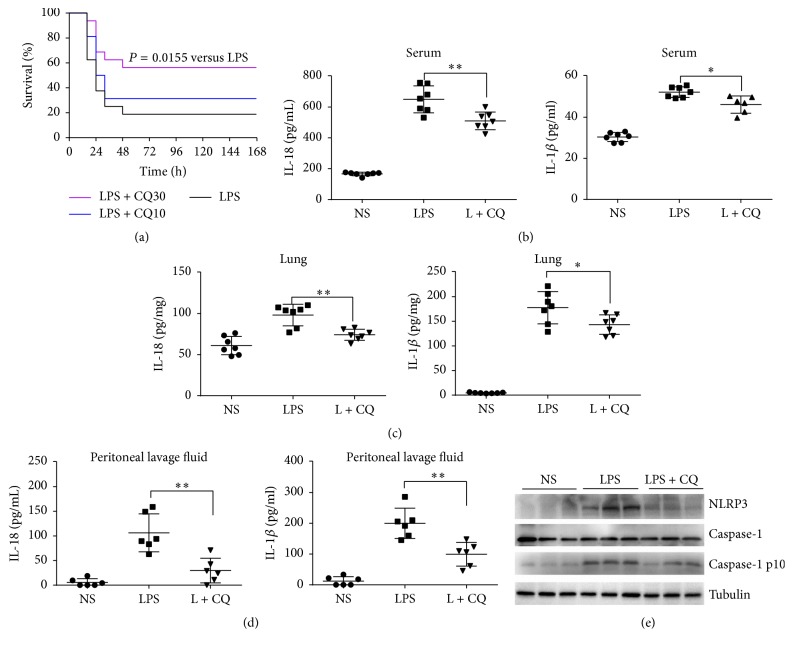
CQ protects mice against lethal endotoxic shock by suppressing NLRP3 inflammasome activation. CQ improved survival in endotoxic shock murine model. BALB/c murine mice (*n* = 16 per group) were injected with a lethal dose of LPS (15 mg/kg) alone or together with CQ (10, 30 mg/kg). (a) Survival in each group was observed for 168 h and the log-rank test was used for intergroup comparison. ^*∗*^*P* < 0.05 versus LPS group. (b) IL-18 and IL-1*β* levels in serum at 6 h were measured by ELISA. (c) IL-18 and IL-1*β* levels in lung homogenates at 6 h were measured by ELISA (*n* = 7 per group). (d) IL-18 and IL-1*β* levels in peritoneal lavage fluid at 6 h were measured by ELISA (*n* = 6 per group). Data are shown as means ± SEM. ^*∗*^*P* < 0.05, ^*∗∗*^*P* < 0.01 versus LPS group. (e) Caspase-1 activation and NLRP3 expression in the lung were detected by western blot (*n* = 3).

We next evaluated the protective effect of CQ against lethal endotoxic shock in mice. For the survival analysis, the survival rate was 18.75% (3/16) in model mice induced by intravenous injection of 15 mg/kg LPS. Administration of CQ (10 and 30 mg/kg) dose-dependently improved the survival rates to 31.25% (5/16) and 56.25% (9/16, *P* = 0.0155), respectively (Figure 6(a)). Serum levels of IL-1*β* and IL-18 were also significantly increased 6 h after LPS injection. Administration of 30 mg/kg CQ markedly attenuated the elevation of IL-1*β* and IL-18 (Figure 6(b)). Similarly, LPS injection caused elevation of IL-1*β* and IL-18 in murine peritoneal lavage fluid and lung homogenates while CQ treatment significantly inhibited the increased effect of LPS (Figures 6(c) and 6(d)). To verify whether CQ could affect NLRP3 inflammasome activation in vivo, we detected NLRP3 and caspase-1 p10 in the lung tissue of LPS challenged murine models. In our study, LPS injection resulted in elevation of NLRP3 and caspase-1 p10 in the lung tissue, indicating the activation of NLRP3 inflammasome in vivo. Treatment with CQ (30 mg/kg) which inhibited IL-1*β* and IL-18 release also inhibited the upregulation of NLRP3 and caspase-1 p10 (Figure 6(e)). Therefore, CQ could block the activation of NLRP3 inflammasome in vivo, which may contribute to the inhibition of IL-1*β* and IL-18 release and improvement of survival in endotoxic shock murine models.

## 4. Discussion

In this study, we illustrated a new role for CQ to significantly dampen the activation of NLRP3 inflammasome and attenuate the production of IL-1*β* and IL-18 upon LPS challenge, which thereby confers protection against lethal endotoxic shock. We further demonstrated that CQ affects NF-*κ*B and MAPK activation and thus suppresses the first signal that induces expression of IL-1*β*, IL-18, and nlrp3. Then, CQ also inhibits the assembly of NLRP3 inflammasome and activation of caspase-1, which resulted in dampened signal 2 for NLRP3 inflammasome activation and maturation/secretion of IL-1*β* and IL-18.

Previously, we found that CQ could interfere with the TLR4 signaling pathway and reduce the production of cytokines like TNF-*α* and IL-6 in monocytes [18, 24]. More recently, CQ was also shown to inhibit IL-1*β* production in human monocytes/macrophages [25]. However, it is not clear how CQ mediates the inhibition. Release of IL-1*β*, as well as its family sibling IL-18, requires activation of TLRs dependent signaling to induce synthesis and activation of inflammasome which catalyzes its maturation and release. Herein, we demonstrated that CQ affected both the intracellular and the extracellular levels of IL-1*β* and IL-18 in LPS treated murine primary macrophages. Such results indicate that CQ not only interferes with the TLR4 signaling pathway but also may possibly target the activation of inflammasomes. Therefore, we compared IL-1*β* inhibition under the condition where CQ was added with LPS priming or ATP treatment alone, to identify which stage may be more essential as its target. Our data demonstrated that cotreatment of CQ with ATP exerts also significantly reduced IL-1*β* release whereas the ability is relatively weaker compared with simultaneous treatment with LPS. Consequently, CQ may be more potent in the transcription stage whereas inhibition of IL-1*β* maturation is newly discovered and also essentially involved in anti-inflammasome activity.

In monocytes/macrophages, LPS induces NF-*κ*B and MAPK pathways activation which act mainly as the first signal for transcriptional regulation of IL-1 and IL-18 production [19, 26, 27]. In the NF-*κ*B pathway, I*κ*B-*α* is phosphorylated to degrade by ubiquitin-dependent proteasomes upon LPS stimulation, which allows NF-*κ*B phosphorylation and disassembly from the heterodimer [28]. In our study, CQ markedly reduced phosphorylation and degradation of I*κ*B-*α* in LPS treated BMDMs, indicating reduced activation and translocation of NF-*κ*B. The p65 protein is a representing subunit of the NF-*κ*B heterodimer. NF-*κ*B p65 is commonly phosphorylated at the serine 536 position upon LPS stimulation and then enters the nucleus to upregulate pro-IL-1*β* and pro-IL-18 [29]. We observed that CQ attenuated LPS-induced p65 phosphorylation (serine 536) and suppressed nucleus translocation of NF-*κ*B p65, which further demonstrated inhibition of the NF-*κ*B signaling pathway. The MAPK pathway also participates in the full activation of NF-*κ*B pathway as well as upregulation of IL-1*β* and IL-18 [30, 31]. Our results also demonstrated that CQ inhibited phosphorylation of ERK1/2, p38, and JNK induced by LPS. Therefore, the above results indicate that pretreatment with CQ inhibits transcriptional upregulation of pro-IL-1*β* and pro-IL-18 through interfering with the NF-*κ*B and MAPK pathway.

NLRP3 is the best characterized inflammasome that cleaves pro-IL-1*β* and pro-IL-18 into their bioactive forms and causes pyroptosis. Activation of NLRP3 inflammasome also requires priming signal (signal 1) and the assembly of NLRP3 inflammasome complex (signal 2). In LPS-primed NLRP3 inflammasome model, the priming signal is the activation of TLR4/NF-*κ*B which leads to elevated expression of NLRP3 and pro-IL-1*β* [32]. In our study, CQ dramatically suppressed NLRP3 expression at both mRNA and protein levels, which is consistent with its effect of inhibiting the expression of IL-1*β* and IL-18. In addition to transcriptional inhibition, active NLRP3 inflammasome leads to the autocleavage of caspase-1 into its active catalyzing form, the caspases-1 p10, which essentially triggers the splicing and maturation of IL-1*β* and IL-18. We also found in this study that pretreatment with CQ inhibited the activity of NLRP3 inflammasome induced by LPS and ATP, which further leads to a reduced level of caspase-1 p10. Active caspase-1 inducing formation of pores and LDH release resulted in the pyroptotic cell death, known as pyroptosis. The dramatically decreased LDH release showed that CQ could inhibit ATP induced pyroptosis in LPS-primed BMDMs. Additionally, multiprotein complex of NLRP3, ASC, and procaspase-1 must be assembled to activate functional NLRP3 inflammasome [32]. ASC nucleation-induced polymerization of oligomerization is considered to be a key event in NLRP3 inflammasome activation [33, 34]. CQ did not change the expression of ASC and procaspase-1 in cell lysates. Instead, CQ inhibited specks formation primed by LPS plus ATP and decreased the percentage of cells containing specks. Recently, potassium efflux out of the cell was unified as a common step that is necessary and sufficient for functional NLRP3 inflammasome [23]. Consistently, prevention of K^+^ efflux dampened the release of IL-1 and enhanced the inhibitory effect of low dose CQ. In our study, CQ also prevented the decline of intracellular K^+^ in response to NLPR3 activators ATP. Taken together, these results showed that CQ inhibits NLRP3 inflammasome activation by decreasing NLRP3 expression, preventing K^+^ efflux, and reducing ASC speck formation. Although the first mechanism (NF-*κ*B) seems to be primary and classic, the second mechanism of NLRP3 inflammasome inhibition may be also essentially involved in anti-inflammasome activity.

Activation of NLRP3 is involved in determining the severity and survival of LPS-induced endotoxin shock in different animal models [35, 36]. As demonstrated by previous studies, LPS-induced endotoxin shock is dampened in NLRP3 deficient murine models, as compared to wild-type murine models. This also suggests that activation of NLRP3 is critical for proinflammatory response and even sepsis in vivo [37]. Previously, CQ was also shown to improve survival and downregulate levels of proinflammatory cytokines in LPS challenged animals [38, 39]. Therefore, we next investigated whether CQ was antagonistic to NLRP3 inflammasome and thus protective in LPS challenged mice models. In our study, CQ could markedly increase the survival of mice by lethal LPS challenge. CQ also effectively reduced IL-1*β* and IL-18 levels in peripheral circulation as well as in peritoneal lavage fluid and major organs severely affected by inflammation, such as the lung. Furthermore, CQ attenuated protein levels of NLRP3 and caspases-1 p10 in the lung tissues, indicating that it exerts consistent activity in the interference with inflammasome activation in vivo, which in turn results in impaired production of IL-1*β* and IL-18. On the other hand, the all-cause death of LPS challenged mice is multifactor-decided and inhibition of each pathogenic factor may contribute at least partly to the overall protection. In the present study, we demonstrated a dual role of CQ in suppressing both NF-*κ*B activation and NLRP3 inflammasome activation. The first effect is more general and contributes to inhibiting production of a wide variety of proinflammatory cytokines [18]. In contrast, the second effect plays more important roles in interfering with the inflammasome associated effect, such as pyroptosis and release of IL-1*β* and IL-18.

## 5. Conclusion

**Figure 7:**
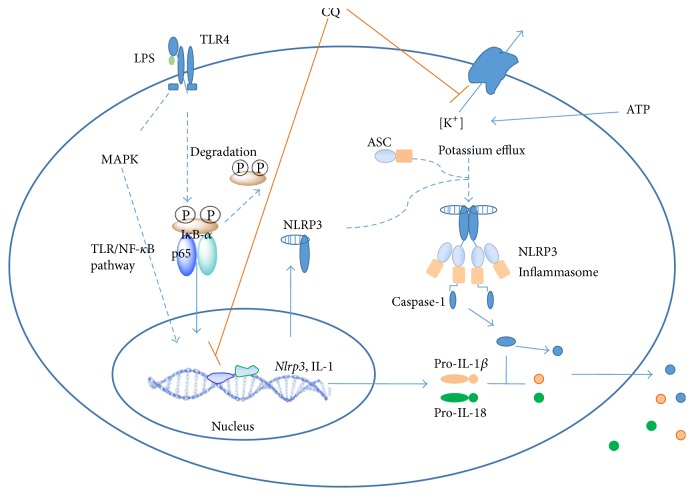
The proposed mechanism of CQ for the inhibition of NLRP3 inflammasome activation.

Taken together, our findings suggest that CQ is a potent inhibitor of the NLRP3 inflammasome pathway. Thereby, it could suppress production of mature IL-1*β* and IL-18 and improve the survival of endotoxic shock by inhibiting NLPR3 inflammasome activation and IL-1*β* and IL-18 maturation. We also identified that the inhibitory effect of CQ on NLRP3 inflammasome activation involves first the suppression on transcription of* nlrp3*, IL-1*β*, and IL-18 by interfering with the NF-*κ*B and MAPK pathway and then the inhibition on assembly of NLRP3 complex by restraining K^+^ efflux and ASC speck formation (Figure 7). These results suggest that CQ might be promising for the therapy of LPS-induced endotoxic shock and other NLRP3 inflammasome related diseases.

## Supplementary Material

The supplementary material includes four figures. CQ inhibits IL-1β secretion in LPS stimulated mouse peritoneal macrophage (Figure S1). CQ inhibits LDH release in LPS-primed BMDMs at different time points (Figure S2). CQ inhibits IL-1β secretion in Pam3CSK4 or R848 stimulated BMDMs (Figure S3). Inhibition of IL-1β secretion in BMDMs by different treatment patterns of CQ (Figure S4).
