# Supplementary material for: The Antimalarial Chloroquine Suppresses LPS-Induced NLRP3 Inflammasome Activation and Confers Protection against Murine Endotoxic Shock
Source: Mediators Inflamm. 2017 Feb 22;2017:6543237. doi: 10.1155/2017/6543237 (PMC5340938; doi:10.1155/2017/6543237)
Supplement: Supplementary file 1 — The supplementary material includes four figures. CQ inhibits IL-1β secretion in LPS stimulated mouse peritoneal macrophage (Figure S1). CQ inhibits LDH release in LPS-primed BMDMs at different time points (Figure S2). CQ inhibits IL-1β secretion in Pam3CSK4 or R848 stimulated BMDMs (Figure S3). Inhibition of IL-1β secretion in BMDMs by different treatment patterns of CQ (Figure S4). [file 6543237.f1.docx]

**Supplementary Materials**





**Figure S1 CQ inhibits IL-1β secretion in LPS stimulated mouse peritoneal macrophage.** Cells were pretreated with indicated concentration of CQ for 1 h, and then primed with LPS (100 ng/ml) for 4 h and ATP (2 mM) was added for additional 1 h treatment. The IL-1β levels in culture supernatants were determined by ELISA (n=3). **: P<0.01 *vs.* LPS plus ATP group.





**Figure S2 CQ inhibits LDH release in BMDMs treated by LPS and ATP.** BMDMs were pretreated with indicated concentration of CQ for 1 h, and then primed with LPS (100 ng/ml) for 4 h and ATP (2 mM) was added for additional 1h, 2h, or 4h treatment. The LDH release in supernatant was detected by LDH assay (n=4). **: P<0.01 *vs.* LPS plus ATP group.





**Figure S3 CQ inhibits IL-1β secretion in Pam3CSK4 or R848 stimulated BMDMs.** BMDMs were pretreated with indicated concentration of CQ for 1 h, and then primed with Pam3CSK4 (500 ng/mL)or R848(2 μg/mL) for 4 h and ATP (2 mM) was added for additional 1 h treatment. The IL-1β levels in culture supernatants were determined by ELISA (n=4). **: P<0.01 *vs.* Pam3CSK4 plus ATP group, ##: P<0.01 *vs.* R848 plus ATP group.

**

**

**Figure S4 Inhibition of IL-1β secretion in LPS and ATP stimulated BMDMs by different treatment patterns of CQ. (A)** BMDMs were pretreated with indicated concentration of CQ for 1 h, primed with LPS (100 ng/ml) for 4 h and treated with ATP (2 mM) for additional 1 h treatment (pretreated). BMDMs were also primed with LPS and then co-treated with ATP and indicated concentration of CQ for 1h (co-treated). The IL-1β levels in culture supernatants were determined by ELISA (n=4). **: P<0.01 *vs.* LPS plus ATP group, ##: P<0.01 *vs* CQ pretreated group. (B) LPS-primed BMDMs were pretreated with indicated concentration of CQ for 1 h. Then CQ was either maintained or removed by cell wash before adding ATP (2 mM) for additional 1 h. The IL-1β levels in culture supernatants were determined by ELISA (n=4). **: P<0.01 *vs*. LPS plus ATP group, ##: P<0.01 *vs*. LPS plus ATP (wash).
